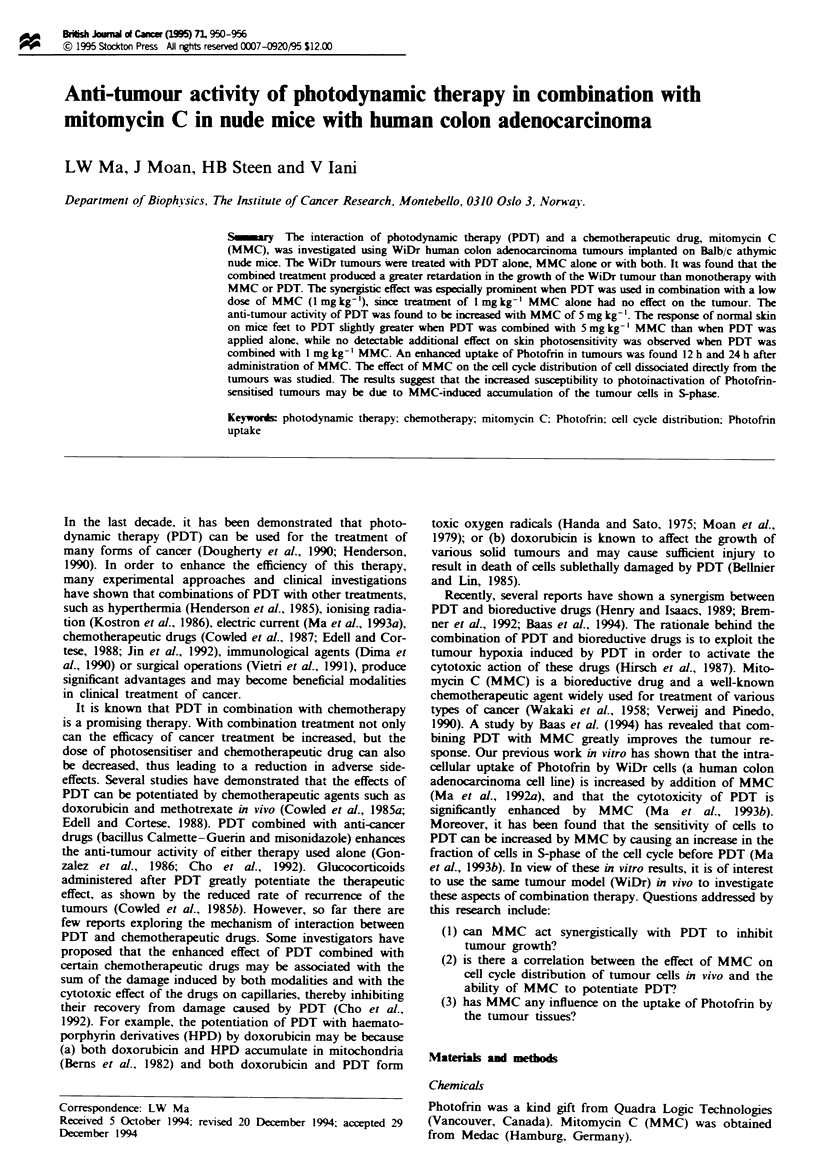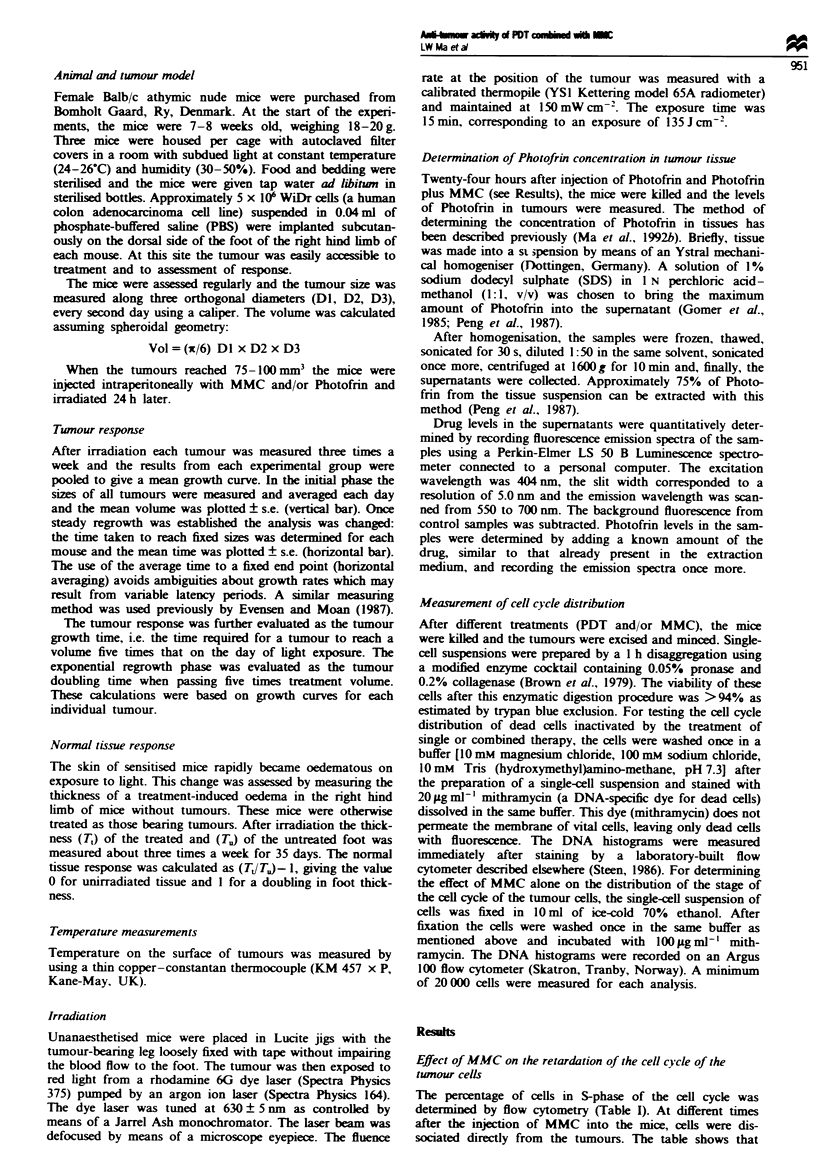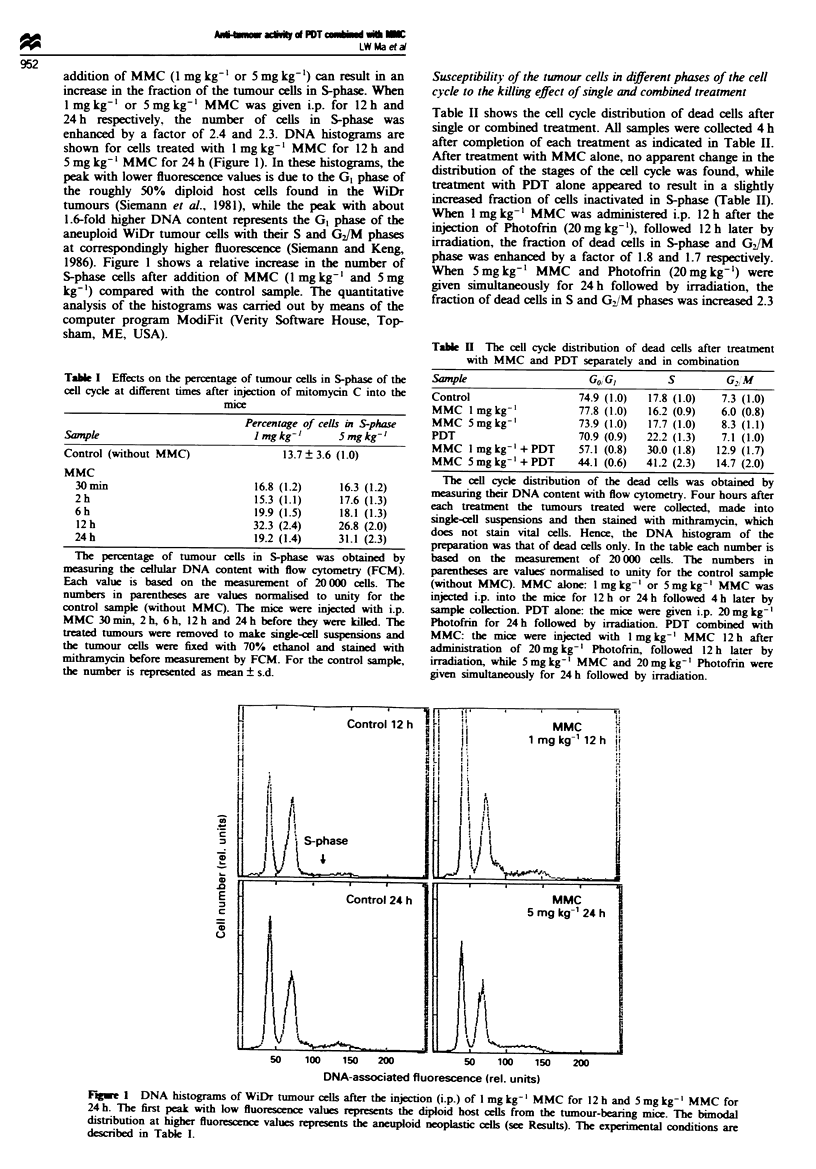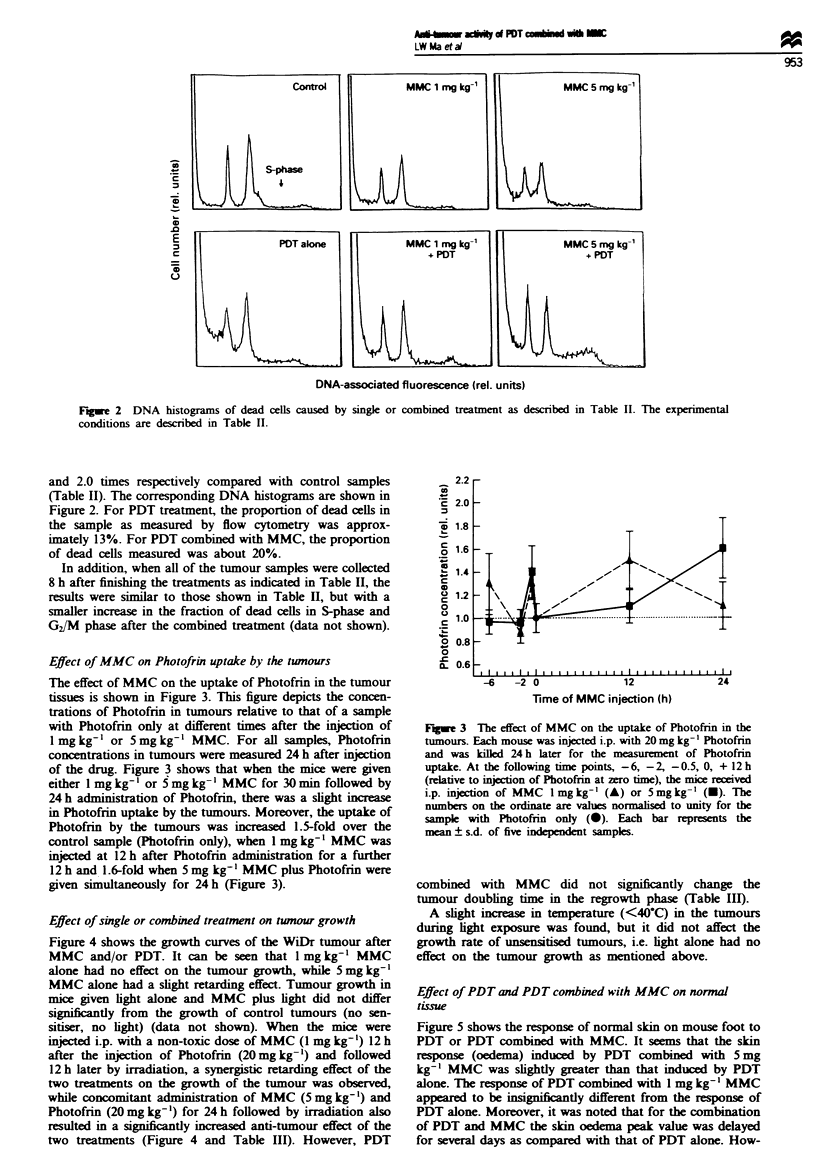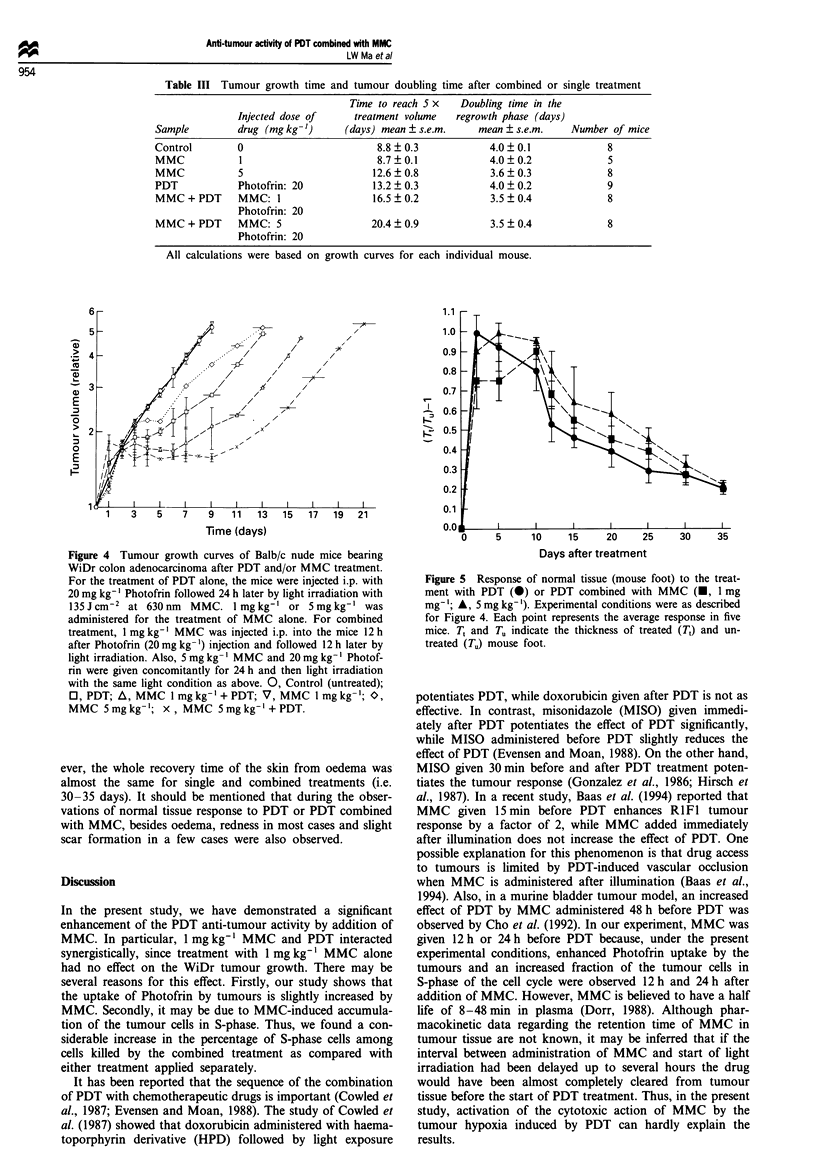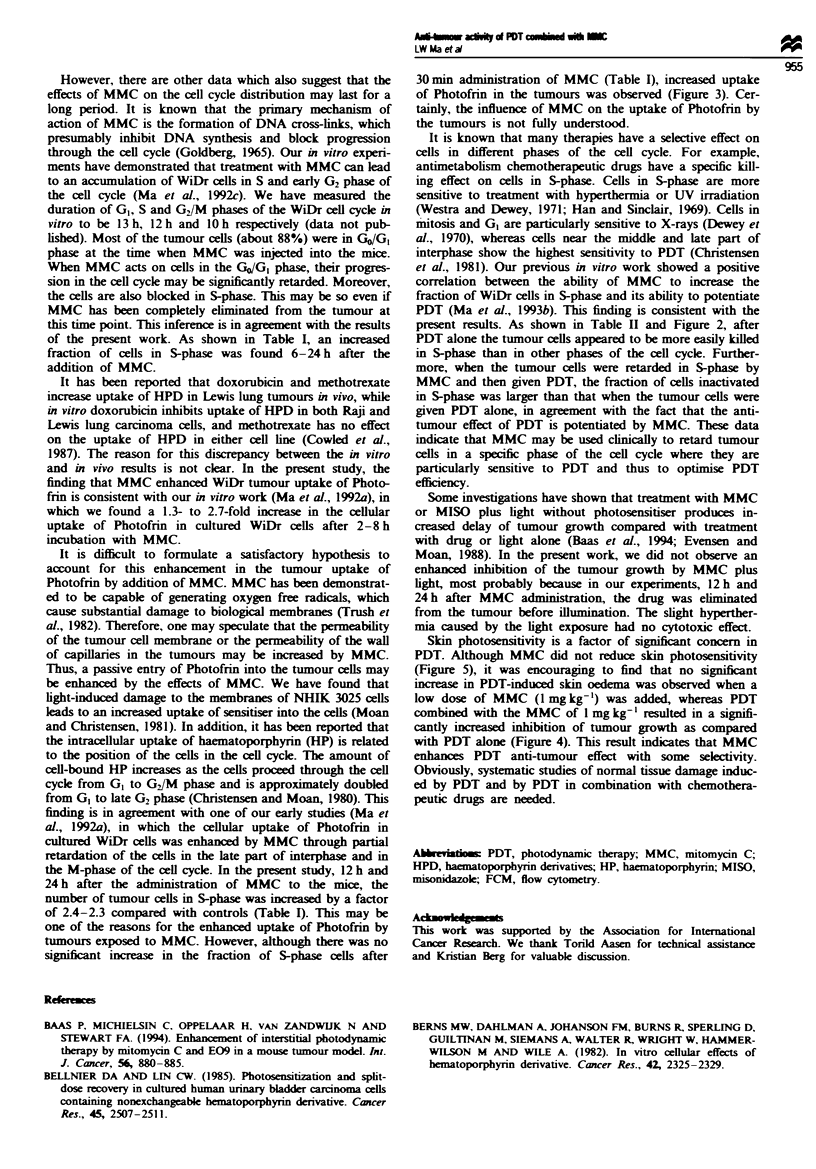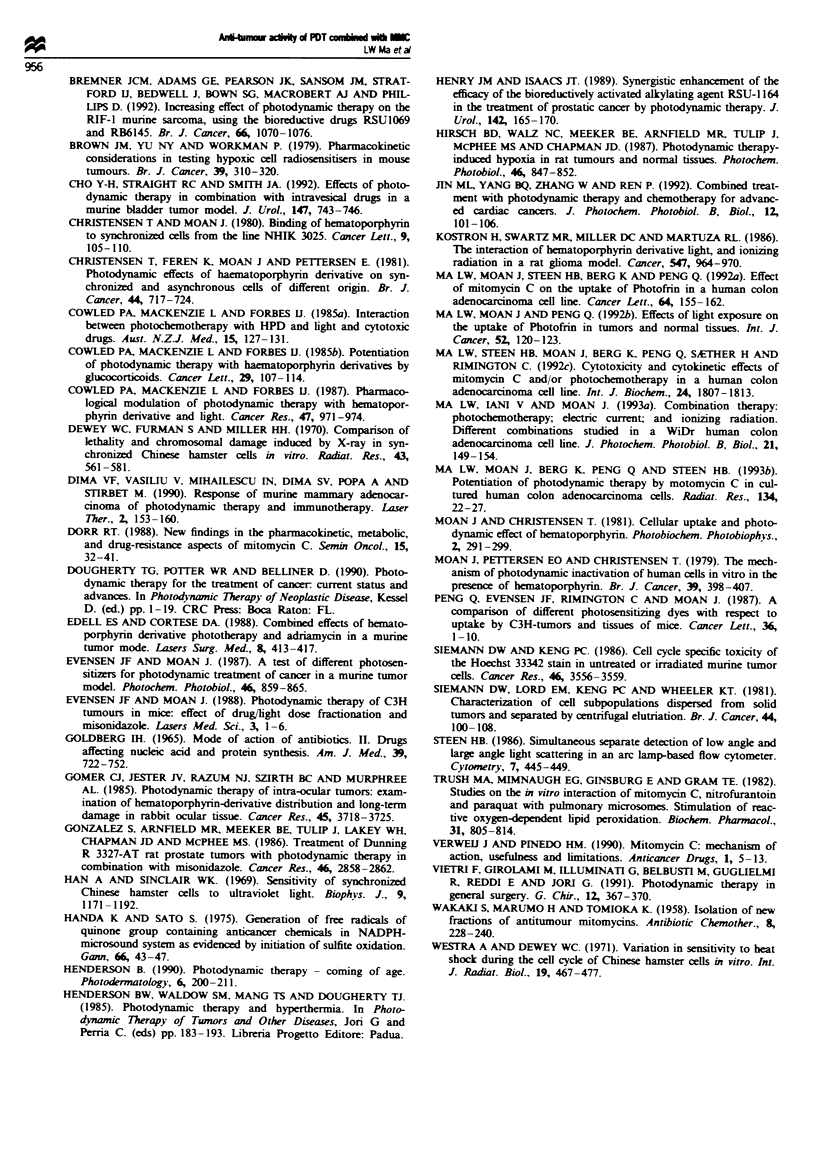# Anti-tumour activity of photodynamic therapy in combination with mitomycin C in nude mice with human colon adenocarcinoma.

**DOI:** 10.1038/bjc.1995.184

**Published:** 1995-05

**Authors:** L. W. Ma, J. Moan, H. B. Steen, V. Iani

**Affiliations:** Department of Biophysics, Institute of Cancer Research, Montebello, Oslo, Norway.

## Abstract

The interaction of photodynamic therapy (PDT) and a chemotherapeutic drug, mitomycin C (MMC), was investigated using WiDr human colon adenocarcinoma tumours implanted on Balb/c athymic nude mice. The WiDr tumours were treated with PDT alone, MMC alone or with both. It was found that the combined treatment produced a greater retardation in the growth of the WiDr tumour than monotherapy with MMC or PDT. The synergistic effect was especially prominent when PDT was used in combination with a low dose of MMC (1 mg kg-1), since treatment of 1 mg kg-1 MMC alone had no effect on the tumour. The anti-tumour activity of PDT was found to be increased with MMC of 5 mg kg-1. The response of normal skin on mice feet to PDT slightly greater when PDT was combined with 5 mg kg-1 MMC than when PDT was applied alone, while no detectable additional effect on skin photosensitivity was observed when PDT was combined with 1 mg kg-1 MMC. An enhanced uptake of Photofrin in tumours was found 12 h and 24 h after administration of MMC. The effect of MMC on the cell cycle distribution of cell dissociated directly from the tumours was studied. The results suggest that the increased susceptibility to photoinactivation of Photofrin-sensitised tumours may be due to MMC-induced accumulation of the tumour cells in S-phase.


British Journal of Cancer (1995) 71, 950-956

© 1995 Stockton Press All rights reserved 0007-0920/95 $12.00

### Anti-tumour activity of photodynamic therapy in combination with mitomycin C in nude mice with human colon adenocarcinoma

LW Ma, J Moan, HB Steen and V Iani

*Department of Biophysics, The Institute of Cancer Research, Montebello, 0310 Oslo 3, Norway.*

**Summary** The interaction of photodynamic therapy (PDT) and a chemotherapeutic drug, mitomycin C (MMC), was investigated using WiDr human colon adenocarcinoma tumours implanted on Balb/c athymic nude mice. The WiDr tumours were treated with PDT alone, MMC alone or with both. It was found that the combined treatment produced a greater retardation in the growth of the WiDr tumour than monotherapy with MMC or PDT. The synergistic effect was especially prominent when PDT was used in combination with a low dose of MMC (1 mg kg$^{-1}$), since treatment of 1 mg kg$^{-1}$ MMC alone had no effect on the tumour. The anti-tumour activity of PDT was found to be increased with MMC of 5 mg kg$^{-1}$. The response of normal skin on mice feet to PDT slightly greater when PDT was combined with 5 mg kg$^{-1}$ MMC than when PDT was applied alone, while no detectable additional effect on skin photosensitivity was observed when PDT was combined with 1 mg kg$^{-1}$ MMC. An enhanced uptake of Photofrin in tumours was found 12 h and 24 h after administration of MMC. The effect of MMC on the cell cycle distribution of cell dissociated directly from the tumours was studied. The results suggest that the increased susceptibility to photoinactivation of Photofrin-sensitised tumours may be due to MMC-induced accumulation of the tumour cells in S-phase.

**Keywords:** photodynamic therapy; chemotherapy; mitomycin C; Photofrin; cell cycle distribution; Photofrin uptake

In the last decade, it has been demonstrated that photodynamic therapy (PDT) can be used for the treatment of many forms of cancer (Dougherty *et al.*, 1990; Henderson, 1990). In order to enhance the efficiency of this therapy, many experimental approaches and clinical investigations have shown that combinations of PDT with other treatments, such as hyperthermia (Henderson *et al.*, 1985), ionising radiation (Kostron *et al.*, 1986), electric current (Ma *et al.*, 1993*a*), chemotherapeutic drugs (Cowled *et al.*, 1987; Edell and Cortese, 1988; Jin *et al.*, 1992), immunological agents (Dima *et al.*, 1990) or surgical operations (Vietri *et al.*, 1991), produce significant advantages and may become beneficial modalities in clinical treatment of cancer.

It is known that PDT in combination with chemotherapy is a promising therapy. With combination treatment not only can the efficacy of cancer treatment be increased, but the dose of photosensitiser and chemotherapeutic drug can also be decreased, thus leading to a reduction in adverse side-effects. Several studies have demonstrated that the effects of PDT can be potentiated by chemotherapeutic agents such as doxorubicin and methotrexate *in vivo* (Cowled *et al.*, 1985*a*; Edell and Cortese, 1988). PDT combined with anti-cancer drugs (bacillus Calmette-Guerin and misonidazole) enhances the anti-tumour activity of either therapy used alone (Gonzalez *et al.*, 1986; Cho *et al.*, 1992). Glucocorticoids administered after PDT greatly potentiate the therapeutic effect, as shown by the reduced rate of recurrence of the tumours (Cowled *et al.*, 1985*b*). However, so far there are few reports exploring the mechanism of interaction between PDT and chemotherapeutic drugs. Some investigators have proposed that the enhanced effect of PDT combined with certain chemotherapeutic drugs may be associated with the sum of the damage induced by both modalities and with the cytotoxic effect of the drugs on capillaries, thereby inhibiting their recovery from damage caused by PDT (Cho *et al.*, 1992). For example, the potentiation of PDT with haematoporphyrin derivatives (HPD) by doxorubicin may be because (a) both doxorubicin and HPD accumulate in mitochondria (Berns *et al.*, 1982) and both doxorubicin and PDT form toxic oxygen radicals (Handa and Sato, 1975; Moan *et al.*, 1979); or (b) doxorubicin is known to affect the growth of various solid tumours and may cause sufficient injury to result in death of cells sublethally damaged by PDT (Bellnier and Lin, 1985).

Recently, several reports have shown a synergism between PDT and bioreductive drugs (Henry and Isaacs, 1989; Bremner *et al.*, 1992; Baas *et al.*, 1994). The rationale behind the combination of PDT and bioreductive drugs is to exploit the tumour hypoxia induced by PDT in order to activate the cytotoxic action of these drugs (Hirsch *et al.*, 1987). Mitomycin C (MMC) is a bioreductive drug and a well-known chemotherapeutic agent widely used for treatment of various types of cancer (Wakaki *et al.*, 1958; Verweij and Pinedo, 1990). A study by Baas *et al.* (1994) has revealed that combining PDT with MMC greatly improves the tumour response. Our previous work *in vitro* has shown that the intracellular uptake of Photofrin by WiDr cells (a human colon adenocarcinoma cell line) is increased by addition of MMC (Ma *et al.*, 1992*a*), and that the cytotoxicity of PDT is significantly enhanced by MMC (Ma *et al.*, 1993*b*). Moreover, it has been found that the sensitivity of cells to PDT can be increased by MMC by causing an increase in the fraction of cells in S-phase of the cell cycle before PDT (Ma *et al.*, 1993*b*). In view of these *in vitro* results, it is of interest to use the same tumour model (WiDr) *in vivo* to investigate these aspects of combination therapy. Questions addressed by this research include:

(1) can MMC act synergistically with PDT to inhibit tumour growth?
(2) is there a correlation between the effect of MMC on cell cycle distribution of tumour cells *in vivo* and the ability of MMC to potentiate PDT?
(3) has MMC any influence on the uptake of Photofrin by the tumour tissues?

### Materials and methods

### *Chemicals*

Photofrin was a kind gift from Quadra Logic Technologies (Vancouver, Canada). Mitomycin C (MMC) was obtained from Medac (Hamburg, Germany).

Correspondence: LW Ma
Received 5 October 1994; revised 20 December 1994; accepted 29 December 1994

*Animal and tumour model*

Female Balb/c athymic nude mice were purchased from Bomholt Gaard, Ry, Denmark. At the start of the experiments, the mice were 7–8 weeks old, weighing 18–20 g. Three mice were housed per cage with autoclaved filter covers in a room with subdued light at constant temperature (24–26°C) and humidity (30–50%). Food and bedding were sterilised and the mice were given tap water *ad libitum* in sterilised bottles. Approximately $5 \times 10^6$ WiDr cells (a human colon adenocarcinoma cell line) suspended in 0.04 ml of phosphate-buffered saline (PBS) were implanted subcutaneously on the dorsal side of the foot of the right hind limb of each mouse. At this site the tumour was easily accessible to treatment and to assessment of response.

The mice were assessed regularly and the tumour size was measured along three orthogonal diameters (D1, D2, D3), every second day using a caliper. The volume was calculated assuming spheroidal geometry:

$$\mathrm{Vol} = (\pi/6)\ \mathrm{D1} \times \mathrm{D2} \times \mathrm{D3}$$

When the tumours reached 75–100 mm³ the mice were injected intraperitoneally with MMC and/or Photofrin and irradiated 24 h later.

*Tumour response*

After irradiation each tumour was measured three times a week and the results from each experimental group were pooled to give a mean growth curve. In the initial phase the sizes of all tumours were measured and averaged each day and the mean volume was plotted ± s.e. (vertical bar). Once steady regrowth was established the analysis was changed: the time taken to reach fixed sizes was determined for each mouse and the mean time was plotted ± s.e. (horizontal bar). The use of the average time to a fixed end point (horizontal averaging) avoids ambiguities about growth rates which may result from variable latency periods. A similar measuring method was used previously by Evensen and Moan (1987).

The tumour response was further evaluated as the tumour growth time, i.e. the time required for a tumour to reach a volume five times that on the day of light exposure. The exponential regrowth phase was evaluated as the tumour doubling time when passing five times treatment volume. These calculations were based on growth curves for each individual tumour.

*Normal tissue response*

The skin of sensitised mice rapidly became oedematous on exposure to light. This change was assessed by measuring the thickness of a treatment-induced oedema in the right hind limb of mice without tumours. These mice were otherwise treated as those bearing tumours. After irradiation the thickness ($T_t$) of the treated and ($T_u$) of the untreated foot was measured about three times a week for 35 days. The normal tissue response was calculated as $(T_t/T_u) - 1$, giving the value 0 for unirradiated tissue and 1 for a doubling in foot thickness.

*Temperature measurements*

Temperature on the surface of tumours was measured by using a thin copper–constantan thermocouple (KM 457 × P, Kane-May, UK).

*Irradiation*

Unanaesthetised mice were placed in Lucite jigs with the tumour-bearing leg loosely fixed with tape without impairing the blood flow to the foot. The tumour was then exposed to red light from a rhodamine 6G dye laser (Spectra Physics 375) pumped by an argon ion laser (Spectra Physics 164). The dye laser was tuned at 630 ± 5 nm as controlled by means of a Jarrel Ash monochromator. The laser beam was defocused by means of a microscope eyepiece. The fluence rate at the position of the tumour was measured with a calibrated thermopile (YSI Kettering model 65A radiometer) and maintained at 150 mW cm$^{-2}$. The exposure time was 15 min, corresponding to an exposure of 135 J cm$^{-2}$.

*Determination of Photofrin concentration in tumour tissue*

Twenty-four hours after injection of Photofrin and Photofrin plus MMC (see Results), the mice were killed and the levels of Photofrin in tumours were measured. The method of determining the concentration of Photofrin in tissues has been described previously (Ma *et al.*, 1992*b*). Briefly, tissue was made into a suspension by means of an Ystral mechanical homogeniser (Dottingen, Germany). A solution of 1% sodium dodecyl sulphate (SDS) in 1 N perchloric acid–methanol (1:1, v/v) was chosen to bring the maximum amount of Photofrin into the supernatant (Gomer *et al.*, 1985; Peng *et al.*, 1987).

After homogenisation, the samples were frozen, thawed, sonicated for 30 s, diluted 1:50 in the same solvent, sonicated once more, centrifuged at 1600 *g* for 10 min and, finally, the supernatants were collected. Approximately 75% of Photofrin from the tissue suspension can be extracted with this method (Peng *et al.*, 1987).

Drug levels in the supernatants were quantitatively determined by recording fluorescence emission spectra of the samples using a Perkin-Elmer LS 50 B Luminescence spectrometer connected to a personal computer. The excitation wavelength was 404 nm, the slit width corresponded to a resolution of 5.0 nm and the emission wavelength was scanned from 550 to 700 nm. The background fluorescence from control samples was subtracted. Photofrin levels in the samples were determined by adding a known amount of the drug, similar to that already present in the extraction medium, and recording the emission spectra once more.

*Measurement of cell cycle distribution*

After different treatments (PDT and/or MMC), the mice were killed and the tumours were excised and minced. Single-cell suspensions were prepared by a 1 h disaggregation using a modified enzyme cocktail containing 0.05% pronase and 0.2% collagenase (Brown *et al.*, 1979). The viability of these cells after this enzymatic digestion procedure was >94% as estimated by trypan blue exclusion. For testing the cell cycle distribution of dead cells inactivated by the treatment of single or combined therapy, the cells were washed once in a buffer [10 mM magnesium chloride, 100 mM sodium chloride, 10 mM Tris (hydroxymethyl)amino-methane, pH 7.3] after the preparation of a single-cell suspension and stained with 20 μg ml$^{-1}$ mithramycin (a DNA-specific dye for dead cells) dissolved in the same buffer. This dye (mithramycin) does not permeate the membrane of vital cells, leaving only dead cells with fluorescence. The DNA histograms were measured immediately after staining by a laboratory-built flow cytometer described elsewhere (Steen, 1986). For determining the effect of MMC alone on the distribution of the stage of the cell cycle of the tumour cells, the single-cell suspension of cells was fixed in 10 ml of ice-cold 70% ethanol. After fixation the cells were washed once in the same buffer as mentioned above and incubated with 100 μg ml$^{-1}$ mithramycin. The DNA histograms were recorded on an Argus 100 flow cytometer (Skatron, Tranby, Norway). A minimum of 20 000 cells were measured for each analysis.

### Results

*Effect of MMC on the retardation of the cell cycle of the tumour cells*

The percentage of cells in S-phase of the cell cycle was determined by flow cytometry (Table I). At different times after the injection of MMC into the mice, cells were dissociated directly from the tumours. The table shows that addition of MMC (1 mg kg$^{-1}$ or 5 mg kg$^{-1}$) can result in an increase in the fraction of the tumour cells in S-phase. When 1 mg kg$^{-1}$ or 5 mg kg$^{-1}$ MMC was given i.p. for 12 h and 24 h respectively, the number of cells in S-phase was enhanced by a factor of 2.4 and 2.3. DNA histograms are shown for cells treated with 1 mg kg$^{-1}$ MMC for 12 h and 5 mg kg$^{-1}$ MMC for 24 h (Figure 1). In these histograms, the peak with lower fluorescence values is due to the $G_1$ phase of the roughly 50% diploid host cells found in the WiDr tumours (Siemann *et al.*, 1981), while the peak with about 1.6-fold higher DNA content represents the $G_1$ phase of the aneuploid WiDr tumour cells with their S and $G_2$/M phases at correspondingly higher fluorescence (Siemann and Keng, 1986). Figure 1 shows a relative increase in the number of S-phase cells after addition of MMC (1 mg kg$^{-1}$ and 5 mg kg$^{-1}$) compared with the control sample. The quantitative analysis of the histograms was carried out by means of the computer program ModiFit (Verity Software House, Topsham, ME, USA).

**Table I** Effects on the percentage of tumour cells in S-phase of the cell cycle at different times after injection of mitomycin C into the mice

| Sample | *Percentage of cells in S-phase* 1 mg kg$^{-1}$ | 5 mg kg$^{-1}$ |
|---|---|---|
| Control (without MMC) | 13.7 ± 3.6 (1.0) | |
| MMC | | |
| 30 min | 16.8 (1.2) | 16.3 (1.2) |
| 2 h | 15.3 (1.1) | 17.6 (1.3) |
| 6 h | 19.9 (1.5) | 18.1 (1.3) |
| 12 h | 32.3 (2.4) | 26.8 (2.0) |
| 24 h | 19.2 (1.4) | 31.1 (2.3) |

The percentage of tumour cells in S-phase was obtained by measuring the cellular DNA content with flow cytometry (FCM). Each value is based on the measurement of 20 000 cells. The numbers in parentheses are values normalised to unity for the control sample (without MMC). The mice were injected with i.p. MMC 30 min, 2 h, 6 h, 12 h and 24 h before they were killed. The treated tumours were removed to make single-cell suspensions and the tumour cells were fixed with 70% ethanol and stained with mithramycin before measurement by FCM. For the control sample, the number is represented as mean ± s.d.

*Susceptibility of the tumour cells in different phases of the cell cycle to the killing effect of single and combined treatment*

Table II shows the cell cycle distribution of dead cells after single or combined treatment. All samples were collected 4 h after completion of each treatment as indicated in Table II. After treatment with MMC alone, no apparent change in the distribution of the stages of the cell cycle was found, while treatment with PDT alone appeared to result in a slightly increased fraction of cells inactivated in S-phase (Table II). When 1 mg kg$^{-1}$ MMC was administered i.p. 12 h after the injection of Photofrin (20 mg kg$^{-1}$), followed 12 h later by irradiation, the fraction of dead cells in S-phase and $G_2$/M phase was enhanced by a factor of 1.8 and 1.7 respectively. When 5 mg kg$^{-1}$ MMC and Photofrin (20 mg kg$^{-1}$) were given simultaneously for 24 h followed by irradiation, the fraction of dead cells in S and $G_2$/M phases was increased 2.3

**Table II** The cell cycle distribution of dead cells after treatment with MMC and PDT separately and in combination

| *Sample* | $G_0/G_1$ | *S* | $G_2/M$ |
|---|---|---|---|
| Control | 74.9 (1.0) | 17.8 (1.0) | 7.3 (1.0) |
| MMC 1 mg kg$^{-1}$ | 77.8 (1.0) | 16.2 (0.9) | 6.0 (0.8) |
| MMC 5 mg kg$^{-1}$ | 73.9 (1.0) | 17.7 (1.0) | 8.3 (1.1) |
| PDT | 70.9 (0.9) | 22.2 (1.3) | 7.1 (1.0) |
| MMC 1 mg kg$^{-1}$ + PDT | 57.1 (0.8) | 30.0 (1.8) | 12.9 (1.7) |
| MMC 5 mg kg$^{-1}$ + PDT | 44.1 (0.6) | 41.2 (2.3) | 14.7 (2.0) |

The cell cycle distribution of the dead cells was obtained by measuring their DNA content with flow cytometry. Four hours after each treatment the tumours treated were collected, made into single-cell suspensions and then stained with mithramycin, which does not stain vital cells. Hence, the DNA histogram of the preparation was that of dead cells only. In the table each number is based on the measurement of 20 000 cells. The numbers in parentheses are values normalised to unity for the control sample (without MMC). MMC alone: 1 mg kg$^{-1}$ or 5 mg kg$^{-1}$ MMC was injected i.p. into the mice for 12 h or 24 h followed 4 h later by sample collection. PDT alone: the mice were given i.p. 20 mg kg$^{-1}$ Photofrin for 24 h followed by irradiation. PDT combined with MMC: the mice were injected with 1 mg kg$^{-1}$ MMC 12 h after administration of 20 mg kg$^{-1}$ Photofrin, followed 12 h later by irradiation, while 5 mg kg$^{-1}$ MMC and 20 mg kg$^{-1}$ Photofrin were given simultaneously for 24 h followed by irradiation.

**Figure 1** DNA histograms of WiDr tumour cells after the injection (i.p.) of 1 mg kg$^{-1}$ MMC for 12 h and 5 mg kg$^{-1}$ MMC for 24 h. The first peak with low fluorescence values represents the diploid host cells from the tumour-bearing mice. The bimodal distribution at higher fluorescence values represents the aneuploid neoplastic cells (see Results). The experimental conditions are described in Table I.

**Figure 2** DNA histograms of dead cells caused by single or combined treatment as described in Table II. The experimental conditions are described in Table II.

and 2.0 times respectively compared with control samples (Table II). The corresponding DNA histograms are shown in Figure 2. For PDT treatment, the proportion of dead cells in the sample as measured by flow cytometry was approximately 13%. For PDT combined with MMC, the proportion of dead cells measured was about 20%.

In addition, when all of the tumour samples were collected 8 h after finishing the treatments as indicated in Table II, the results were similar to those shown in Table II, but with a smaller increase in the fraction of dead cells in S-phase and $G_2/M$ phase after the combined treatment (data not shown).

*Effect of MMC on Photofrin uptake by the tumours*

The effect of MMC on the uptake of Photofrin in the tumour tissues is shown in Figure 3. This figure depicts the concentrations of Photofrin in tumours relative to that of a sample with Photofrin only at different times after the injection of 1 mg kg$^{-1}$ or 5 mg kg$^{-1}$ MMC. For all samples, Photofrin concentrations in tumours were measured 24 h after injection of the drug. Figure 3 shows that when the mice were given either 1 mg kg$^{-1}$ or 5 mg kg$^{-1}$ MMC for 30 min followed by 24 h administration of Photofrin, there was a slight increase in Photofrin uptake by the tumours. Moreover, the uptake of Photofrin by the tumours was increased 1.5-fold over the control sample (Photofrin only), when 1 mg kg$^{-1}$ MMC was injected at 12 h after Photofrin administration for a further 12 h and 1.6-fold when 5 mg kg$^{-1}$ MMC plus Photofrin were given simultaneously for 24 h (Figure 3).

**Figure 3** The effect of MMC on the uptake of Photofrin in the tumours. Each mouse was injected i.p. with 20 mg kg$^{-1}$ Photofrin and was killed 24 h later for the measurement of Photofrin uptake. At the following time points, −6, −2, −0.5, 0, + 12 h (relative to injection of Photofrin at zero time), the mice received i.p. injection of MMC 1 mg kg$^{-1}$ (▲) or 5 mg kg$^{-1}$ (■). The numbers on the ordinate are values normalised to unity for the sample with Photofrin only (●). Each bar represents the mean ± s.d. of five independent samples.

*Effect of single or combined treatment on tumour growth*

Figure 4 shows the growth curves of the WiDr tumour after MMC and/or PDT. It can be seen that 1 mg kg$^{-1}$ MMC alone had no effect on the tumour growth, while 5 mg kg$^{-1}$ MMC alone had a slight retarding effect. Tumour growth in mice given light alone and MMC plus light did not differ significantly from the growth of control tumours (no sensitiser, no light) (data not shown). When the mice were injected i.p. with a non-toxic dose of MMC (1 mg kg$^{-1}$) 12 h after the injection of Photofrin (20 mg kg$^{-1}$) and followed 12 h later by irradiation, a synergistic retarding effect of the two treatments on the growth of the tumour was observed, while concomitant administration of MMC (5 mg kg$^{-1}$) and Photofrin (20 mg kg$^{-1}$) for 24 h followed by irradiation also resulted in a significantly increased anti-tumour effect of the two treatments (Figure 4 and Table III). However, PDT combined with MMC did not significantly change the tumour doubling time in the regrowth phase (Table III).

A slight increase in temperature (<40°C) in the tumours during light exposure was found, but it did not affect the growth rate of unsensitised tumours, i.e. light alone had no effect on the tumour growth as mentioned above.

*Effect of PDT and PDT combined with MMC on normal tissue*

Figure 5 shows the response of normal skin on mouse foot to PDT or PDT combined with MMC. It seems that the skin response (oedema) induced by PDT combined with 5 mg kg$^{-1}$ MMC was slightly greater than that induced by PDT alone. The response of PDT combined with 1 mg kg$^{-1}$ MMC appeared to be insignificantly different from the response of PDT alone. Moreover, it was noted that for the combination of PDT and MMC the skin oedema peak value was delayed for several days as compared with that of PDT alone. How-

**Table III** Tumour growth time and tumour doubling time after combined or single treatment

| *Sample* | *Injected dose of drug (mg kg⁻¹)* | *Time to reach 5 × treatment volume (days) mean ± s.e.m.* | *Doubling time in the regrowth phase (days) mean ± s.e.m.* | *Number of mice* |
|---|---|---|---|---|
| Control | 0 | 8.8 ± 0.3 | 4.0 ± 0.1 | 8 |
| MMC | 1 | 8.7 ± 0.1 | 4.0 ± 0.2 | 5 |
| MMC | 5 | 12.6 ± 0.8 | 3.6 ± 0.3 | 8 |
| PDT | Photofrin: 20 | 13.2 ± 0.3 | 4.0 ± 0.2 | 9 |
| MMC + PDT | MMC: 1 Photofrin: 20 | 16.5 ± 0.2 | 3.5 ± 0.4 | 8 |
| MMC + PDT | MMC: 5 Photofrin: 20 | 20.4 ± 0.9 | 3.5 ± 0.4 | 8 |

All calculations were based on growth curves for each individual mouse.

**Figure 4** Tumour growth curves of Balb/c nude mice bearing WiDr colon adenocarcinoma after PDT and/or MMC treatment. For the treatment of PDT alone, the mice were injected i.p. with 20 mg kg$^{-1}$ Photofrin followed 24 h later by light irradiation with 135 J cm$^{-2}$ at 630 nm MMC. 1 mg kg$^{-1}$ or 5 mg kg$^{-1}$ was administered for the treatment of MMC alone. For combined treatment, 1 mg kg$^{-1}$ MMC was injected i.p. into the mice 12 h after Photofrin (20 mg kg$^{-1}$) injection and followed 12 h later by light irradiation. Also, 5 mg kg$^{-1}$ MMC and 20 mg kg$^{-1}$ Photofrin were given concomitantly for 24 h and then light irradiation with the same light condition as above. ○, Control (untreated); □, PDT; △, MMC 1 mg kg$^{-1}$ + PDT; ▽, MMC 1 mg kg$^{-1}$; ◇, MMC 5 mg kg$^{-1}$; ×, MMC 5 mg kg$^{-1}$ + PDT.

**Figure 5** Response of normal tissue (mouse foot) to the treatment with PDT (●) or PDT combined with MMC (■, 1 mg mg$^{-1}$; ▲, 5 mg kg$^{-1}$). Experimental conditions were as described for Figure 4. Each point represents the average response in five mice. $T_t$ and $T_u$ indicate the thickness of treated ($T_t$) and untreated ($T_u$) mouse foot.

ever, the whole recovery time of the skin from oedema was almost the same for single and combined treatments (i.e. 30–35 days). It should be mentioned that during the observations of normal tissue response to PDT or PDT combined with MMC, besides oedema, redness in most cases and slight scar formation in a few cases were also observed.

### Discussion

In the present study, we have demonstrated a significant enhancement of the PDT anti-tumour activity by addition of MMC. In particular, 1 mg kg$^{-1}$ MMC and PDT interacted synergistically, since treatment with 1 mg kg$^{-1}$ MMC alone had no effect on the WiDr tumour growth. There may be several reasons for this effect. Firstly, our study shows that the uptake of Photofrin by tumours is slightly increased by MMC. Secondly, it may be due to MMC-induced accumulation of the tumour cells in S-phase. Thus, we found a considerable increase in the percentage of S-phase cells among cells killed by the combined treatment as compared with either treatment applied separately.

It has been reported that the sequence of the combination of PDT with chemotherapeutic drugs is important (Cowled *et al.*, 1987; Evensen and Moan, 1988). The study of Cowled *et al.* (1987) showed that doxorubicin administered with haematoporphyrin derivative (HPD) followed by light exposure potentiates PDT, while doxorubicin given after PDT is not as effective. In contrast, misonidazole (MISO) given immediately after PDT potentiates the effect of PDT significantly, while MISO administered before PDT slightly reduces the effect of PDT (Evensen and Moan, 1988). On the other hand, MISO given 30 min before and after PDT treatment potentiates the tumour response (Gonzalez *et al.*, 1986; Hirsch *et al.*, 1987). In a recent study, Baas *et al.* (1994) reported that MMC given 15 min before PDT enhances R1F1 tumour response by a factor of 2, while MMC added immediately after illumination does not increase the effect of PDT. One possible explanation for this phenomenon is that drug access to tumours is limited by PDT-induced vascular occlusion when MMC is administered after illumination (Baas *et al.*, 1994). Also, in a murine bladder tumour model, an increased effect of PDT by MMC administered 48 h before PDT was observed by Cho *et al.* (1992). In our experiment, MMC was given 12 h or 24 h before PDT because, under the present experimental conditions, enhanced Photofrin uptake by the tumours and an increased fraction of the tumour cells in S-phase of the cell cycle were observed 12 h and 24 h after addition of MMC. However, MMC is believed to have a half life of 8–48 min in plasma (Dorr, 1988). Although pharmacokinetic data regarding the retention time of MMC in tumour tissue are not known, it may be inferred that if the interval between administration of MMC and start of light irradiation had been delayed up to several hours the drug would have been almost completely cleared from tumour tissue before the start of PDT treatment. Thus, in the present study, activation of the cytotoxic action of MMC by the tumour hypoxia induced by PDT can hardly explain the results.

However, there are other data which also suggest that the effects of MMC on the cell cycle distribution may last for a long period. It is known that the primary mechanism of action of MMC is the formation of DNA cross-links, which presumably inhibit DNA synthesis and block progression through the cell cycle (Goldberg, 1965). Our *in vitro* experiments have demonstrated that treatment with MMC can lead to an accumulation of WiDr cells in S and early $G_2$ phase of the cell cycle (Ma *et al.*, 1992*c*). We have measured the duration of $G_1$, S and $G_2$/M phases of the WiDr cell cycle *in vitro* to be 13 h, 12 h and 10 h respectively (data not published). Most of the tumour cells (about 88%) were in $G_0/G_1$ phase at the time when MMC was injected into the mice. When MMC acts on cells in the $G_0/G_1$ phase, their progression in the cell cycle may be significantly retarded. Moreover, the cells are also blocked in S-phase. This may be so even if MMC has been completely eliminated from the tumour at this time point. This inference is in agreement with the results of the present work. As shown in Table I, an increased fraction of cells in S-phase was found 6–24 h after the addition of MMC.

It has been reported that doxorubicin and methotrexate increase uptake of HPD in Lewis lung tumours *in vivo*, while *in vitro* doxorubicin inhibits uptake of HPD in both Raji and Lewis lung carcinoma cells, and methotrexate has no effect on the uptake of HPD in either cell line (Cowled *et al.*, 1987). The reason for this discrepancy between the *in vitro* and *in vivo* results is not clear. In the present study, the finding that MMC enhanced WiDr tumour uptake of Photofrin is consistent with our *in vitro* work (Ma *et al.*, 1992*a*), in which we found a 1.3- to 2.7-fold increase in the cellular uptake of Photofrin in cultured WiDr cells after 2–8 h incubation with MMC.

It is difficult to formulate a satisfactory hypothesis to account for this enhancement in the tumour uptake of Photofrin by addition of MMC. MMC has been demonstrated to be capable of generating oxygen free radicals, which cause substantial damage to biological membranes (Trush *et al.*, 1982). Therefore, one may speculate that the permeability of the tumour cell membrane or the permeability of the wall of capillaries in the tumours may be increased by MMC. Thus, a passive entry of Photofrin into the tumour cells may be enhanced by the effects of MMC. We have found that light-induced damage to the membranes of NHIK 3025 cells leads to an increased uptake of sensitiser into the cells (Moan and Christensen, 1981). In addition, it has been reported that the intracellular uptake of haematoporphyrin (HP) is related to the position of the cells in the cell cycle. The amount of cell-bound HP increases as the cells proceed through the cell cycle from $G_1$ to $G_2$/M phase and is approximately doubled from $G_1$ to late $G_2$ phase (Christensen and Moan, 1980). This finding is in agreement with one of our early studies (Ma *et al.*, 1992*a*), in which the cellular uptake of Photofrin in cultured WiDr cells was enhanced by MMC through partial retardation of the cells in the late part of interphase and in the M-phase of the cell cycle. In the present study, 12 h and 24 h after the administration of MMC to the mice, the number of tumour cells in S-phase was increased by a factor of 2.4–2.3 compared with controls (Table I). This may be one of the reasons for the enhanced uptake of Photofrin by tumours exposed to MMC. However, although there was no significant increase in the fraction of S-phase cells after 30 min administration of MMC (Table I), increased uptake of Photofrin in the tumours was observed (Figure 3). Certainly, the influence of MMC on the uptake of Photofrin by the tumours is not fully understood.

It is known that many therapies have a selective effect on cells in different phases of the cell cycle. For example, antimetabolism chemotherapeutic drugs have a specific killing effect on cells in S-phase. Cells in S-phase are more sensitive to treatment with hyperthermia or UV irradiation (Westra and Dewey, 1971; Han and Sinclair, 1969). Cells in mitosis and $G_1$ are particularly sensitive to X-rays (Dewey *et al.*, 1970), whereas cells near the middle and late part of interphase show the highest sensitivity to PDT (Christensen *et al.*, 1981). Our previous *in vitro* work showed a positive correlation between the ability of MMC to increase the fraction of WiDr cells in S-phase and its ability to potentiate PDT (Ma *et al.*, 1993*b*). This finding is consistent with the present results. As shown in Table II and Figure 2, after PDT alone the tumour cells appeared to be more easily killed in S-phase than in other phases of the cell cycle. Furthermore, when the tumour cells were retarded in S-phase by MMC and then given PDT, the fraction of cells inactivated in S-phase was larger than that when the tumour cells were given PDT alone, in agreement with the fact that the anti-tumour effect of PDT is potentiated by MMC. These data indicate that MMC may be used clinically to retard tumour cells in a specific phase of the cell cycle where they are particularly sensitive to PDT and thus to optimise PDT efficiency.

Some investigations have shown that treatment with MMC or MISO plus light without photosensitiser produces increased delay of tumour growth compared with treatment with drug or light alone (Baas *et al.*, 1994; Evensen and Moan, 1988). In the present work, we did not observe an enhanced inhibition of the tumour growth by MMC plus light, most probably because in our experiments, 12 h and 24 h after MMC administration, the drug was eliminated from the tumour before illumination. The slight hyperthermia caused by the light exposure had no cytotoxic effect.

Skin photosensitivity is a factor of significant concern in PDT. Although MMC did not reduce skin photosensitivity (Figure 5), it was encouraging to find that no significant increase in PDT-induced skin oedema was observed when a low dose of MMC (1 mg $kg^{-1}$) was added, whereas PDT combined with the MMC of 1 mg $kg^{-1}$ resulted in a significantly increased inhibition of tumour growth as compared with PDT alone (Figure 4). This result indicates that MMC enhances PDT anti-tumour effect with some selectivity. Obviously, systematic studies of normal tissue damage induced by PDT and by PDT in combination with chemotherapeutic drugs are needed.

**Abbreviations:** PDT, photodynamic therapy; MMC, mitomycin C; HPD, haematoporphyrin derivatives; HP, haematoporphyrin; MISO, misonidazole; FCM, flow cytometry.

**Acknowledgements**
This work was supported by the Association for International Cancer Research. We thank Torild Aasen for technical assistance and Kristian Berg for valuable discussion.

### References

BAAS P, MICHIELSIN C, OPPELAAR H, VAN ZANDWIJK N AND STEWART FA. (1994). Enhancement of interstitial photodynamic therapy by mitomycin C and EO9 in a mouse tumour model. *Int. J. Cancer*, **56**, 880–885.

BELLNIER DA AND LIN CW. (1985). Photosensitization and split-dose recovery in cultured human urinary bladder carcinoma cells containing nonexchangeable hematoporphyrin derivative. *Cancer Res.*, **45**, 2507–2511.

BERNS MW, DAHLMAN A, JOHANSON FM, BURNS R, SPERLING D, GUILTINAN M, SIEMANS A, WALTER R, WRIGHT W, HAMMER-WILSON M AND WILE A. (1982). In vitro cellular effects of hematoporphyrin derivative. *Cancer Res.*, **42**, 2325–2329.

BREMNER JCM, ADAMS GE, PEARSON JK, SANSOM JM, STRATFORD IJ, BEDWELL J, BOWN SG, MACROBERT AJ AND PHILLIPS D. (1992). Increasing effect of photodynamic therapy on the RIF-1 murine sarcoma, using the bioreductive drugs RSU1069 and RB6145. *Br. J. Cancer*, **66**, 1070–1076.

BROWN JM, YU NY AND WORKMAN P. (1979). Pharmacokinetic considerations in testing hypoxic cell radiosensitisers in mouse tumours. *Br. J. Cancer*, **39**, 310–320.

CHO Y-H, STRAIGHT RC AND SMITH JA. (1992). Effects of photodynamic therapy in combination with intravesical drugs in a murine bladder tumor model. *J. Urol.*, **147**, 743–746.

CHRISTENSEN T AND MOAN J. (1980). Binding of hematoporphyrin to synchronized cells from the line NHIK 3025. *Cancer Lett.*, **9**, 105–110.

CHRISTENSEN T, FEREN K, MOAN J AND PETTERSEN E. (1981). Photodynamic effects of haematoporphyrin derivative on synchronized and asynchronous cells of different origin. *Br. J. Cancer*, **44**, 717–724.

COWLED PA, MACKENZIE L AND FORBES IJ. (1985*a*). Interaction between photochemotherapy with HPD and light and cytotoxic drugs. *Aust. N.Z.J. Med.*, **15**, 127–131.

COWLED PA, MACKENZIE L AND FORBES IJ. (1985*b*). Potentiation of photodynamic therapy with haematoporphyrin derivatives by glucocorticoids. *Cancer Lett.*, **29**, 107–114.

COWLED PA, MACKENZIE L AND FORBES IJ. (1987). Pharmacological modulation of photodynamic therapy with hematoporphyrin derivative and light. *Cancer Res.*, **47**, 971–974.

DEWEY WC, FURMAN S AND MILLER HH. (1970). Comparison of lethality and chromosomal damage induced by X-ray in synchronized Chinese hamster cells *in vitro*. *Radiat. Res.*, **43**, 561–581.

DIMA VF, VASILIU V, MIHAILESCU IN, DIMA SV, POPA A AND STIRBET M. (1990). Response of murine mammary adenocarcinoma of photodynamic therapy and immunotherapy. *Laser Ther.*, **2**, 153–160.

DORR RT. (1988). New findings in the pharmacokinetic, metabolic, and drug-resistance aspects of mitomycin C. *Semin Oncol.*, **15**, 32–41.

DOUGHERTY TG, POTTER WR AND BELLINER D. (1990). Photodynamic therapy for the treatment of cancer: current status and advances. In *Photodynamic Therapy of Neoplastic Disease*, Kessel D. (ed.) pp. 1–19. CRC Press: Boca Raton: FL.

EDELL ES AND CORTESE DA. (1988). Combined effects of hematoporphyrin derivative phototherapy and adriamycin in a murine tumor mode. *Lasers Surg. Med.*, **8**, 413–417.

EVENSEN JF AND MOAN J. (1987). A test of different photosensitizers for photodynamic treatment of cancer in a murine tumor model. *Photochem. Photobiol.*, **46**, 859–865.

EVENSEN JF AND MOAN J. (1988). Photodynamic therapy of C3H tumours in mice: effect of drug/light dose fractionation and misonidazole. *Lasers Med. Sci.*, **3**, 1–6.

GOLDBERG IH. (1965). Mode of action of antibiotics. II. Drugs affecting nucleic acid and protein synthesis. *Am. J. Med.*, **39**, 722–752.

GOMER CJ, JESTER JV, RAZUM NJ, SZIRTH BC AND MURPHREE AL. (1985). Photodynamic therapy of intra-ocular tumors: examination of hematoporphyrin-derivative distribution and long-term damage in rabbit ocular tissue. *Cancer Res.*, **45**, 3718–3725.

GONZALEZ S, ARNFIELD MR, MEEKER BE, TULIP J, LAKEY WH, CHAPMAN JD AND McPHEE MS. (1986). Treatment of Dunning R 3327-AT rat prostate tumors with photodynamic therapy in combination with misonidazole. *Cancer Res.*, **46**, 2858–2862.

HAN A AND SINCLAIR WK. (1969). Sensitivity of synchronized Chinese hamster cells to ultraviolet light. *Biophys. J.*, **9**, 1171–1192.

HANDA K AND SATO S. (1975). Generation of free radicals of quinone group containing anticancer chemicals in NADPH-microsound system as evidenced by initiation of sulfite oxidation. *Gann*, **66**, 43–47.

HENDERSON B. (1990). Photodynamic therapy – coming of age. *Photodermatology*, **6**, 200–211.

HENDERSON BW, WALDOW SM, MANG TS AND DOUGHERTY TJ. (1985). Photodynamic therapy and hyperthermia. In *Photodynamic Therapy of Tumors and Other Diseases*, Jori G and Perria C. (eds) pp. 183–193. Libreria Progetto Editore: Padua.

HENRY JM AND ISAACS JT. (1989). Synergistic enhancement of the efficacy of the bioreductively activated alkylating agent RSU-1164 in the treatment of prostatic cancer by photodynamic therapy. *J. Urol.*, **142**, 165–170.

HIRSCH BD, WALZ NC, MEEKER BE, ARNFIELD MR, TULIP J, McPHEE MS AND CHAPMAN JD. (1987). Photodynamic therapy-induced hypoxia in rat tumours and normal tissues. *Photochem. Photobiol.*, **46**, 847–852.

JIN ML, YANG BQ, ZHANG W AND REN P. (1992). Combined treatment with photodynamic therapy and chemotherapy for advanced cardiac cancers. *J. Photochem. Photobiol. B, Biol.*, **12**, 101–106.

KOSTRON H, SWARTZ MR, MILLER DC AND MARTUZA RL. (1986). The interaction of hematoporphyrin derivative light, and ionizing radiation in a rat glioma model. *Cancer*, **547**, 964–970.

MA LW, MOAN J, STEEN HB, BERG K AND PENG Q. (1992*a*). Effect of mitomycin C on the uptake of Photofrin in a human colon adenocarcinoma cell line. *Cancer Lett.*, **64**, 155–162.

MA LW, MOAN J AND PENG Q. (1992*b*). Effects of light exposure on the uptake of Photofrin in tumors and normal tissues. *Int. J. Cancer*, **52**, 120–123.

MA LW, STEEN HB, MOAN J, BERG K, PENG Q, SÆTHER H AND RIMINGTON C. (1992*c*). Cytotoxicity and cytokinetic effects of mitomycin C and/or photochemotherapy in a human colon adenocarcinoma cell line. *Int. J. Biochem.*, **24**, 1807–1813.

MA LW, IANI V AND MOAN J. (1993*a*). Combination therapy: photochemotherapy; electric current; and ionizing radiation. Different combinations studied in a WiDr human colon adenocarcinoma cell line. *J. Photochem. Photobiol. B, Biol.*, **21**, 149–154.

MA LW, MOAN J, BERG K, PENG Q AND STEEN HB. (1993*b*). Potentiation of photodynamic therapy by motomycin C in cultured human colon adenocarcinoma cells. *Radiat. Res.*, **134**, 22–27.

MOAN J AND CHRISTENSEN T. (1981). Cellular uptake and photodynamic effect of hematoporphyrin. *Photobiochem. Photobiophys.*, **2**, 291–299.

MOAN J, PETTERSEN EO AND CHRISTENSEN T. (1979). The mechanism of photodynamic inactivation of human cells in vitro in the presence of hematoporphyrin. *Br. J. Cancer*, **39**, 398–407.

PENG Q, EVENSEN JF, RIMINGTON C AND MOAN J. (1987). A comparison of different photosensitizing dyes with respect to uptake by C3H-tumors and tissues of mice. *Cancer Lett.*, **36**, 1–10.

SIEMANN DW AND KENG PC. (1986). Cell cycle specific toxicity of the Hoechst 33342 stain in untreated or irradiated murine tumor cells. *Cancer Res.*, **46**, 3556–3559.

SIEMANN DW, LORD EM, KENG PC AND WHEELER KT. (1981). Characterization of cell subpopulations dispersed from solid tumors and separated by centrifugal elutriation. *Br. J. Cancer*, **44**, 100–108.

STEEN HB. (1986). Simultaneous separate detection of low angle and large angle light scattering in an arc lamp-based flow cytometer. *Cytometry*, **7**, 445–449.

TRUSH MA, MIMNAUGH EG, GINSBURG E AND GRAM TE. (1982). Studies on the *in vitro* interaction of mitomycin C, nitrofurantoin and paraquat with pulmonary microsomes. Stimulation of reactive oxygen-dependent lipid peroxidation. *Biochem. Pharmacol.*, **31**, 805–814.

VERWEIJ J AND PINEDO HM. (1990). Mitomycin C: mechanism of action, usefulness and limitations. *Anticancer Drugs*, **1**, 5–13.

VIETRI F, GIROLAMI M, ILLUMINATI G, BELBUSTI M, GUGLIELMI R, REDDI E AND JORI G. (1991). Photodynamic therapy in general surgery. *G. Chir.*, **12**, 367–370.

WAKAKI S, MARUMO H AND TOMIOKA K. (1958). Isolation of new fractions of antitumour mitomycins. *Antibiotic Chemother.*, **8**, 228–240.

WESTRA A AND DEWEY WC. (1971). Variation in sensitivity to heat shock during the cell cycle of Chinese hamster cells *in vitro*. *Int. J. Radiat. Biol.*, **19**, 467–477.